# Butane-1,4-diyl bis­(benzene­carbodi­thio­ate)

**DOI:** 10.1107/S1600536813027529

**Published:** 2013-10-12

**Authors:** Daisuke Abe, Yuji Sasanuma

**Affiliations:** a Department of Applied Chemistry and Biotechnology, Chiba University, 1-33, Yayoi-cho, Inage-ku, Chiba 263-8522, Japan

## Abstract

The title compound, C_18_H_18_S_4_, which lies on an inversion center, adopts a *trans*–*gauche* ^+^–*trans*–*gauche* ^−^–*trans* (*tg* ^+^ *tg* ^−^ *t*) conformation of the S—CH_2_—CH_2_—CH_2_—CH_2_—S bond sequence. In the crystal, a π–π inter­action with a centroid–centroid distance of 3.8797 (16) Å is observed.

## Related literature

For crystal structures and conformations of C_6_H_5_C(=S)S(CH_2_)_2_SC(=S)C_6_H_5_ and C_6_H_5_C(=O)S(CH_2_)_4_SC(=O)C_6_H_5_, see: Abe *et al.* (2011 ▶, 2013 ▶). For related compounds, see: Sawanobori *et al.* (2001 ▶); Sasanuma *et al.* (2002 ▶). For the synthesis of piperidinium di­thio­benzoate, see: Kato *et al.* (1973 ▶).
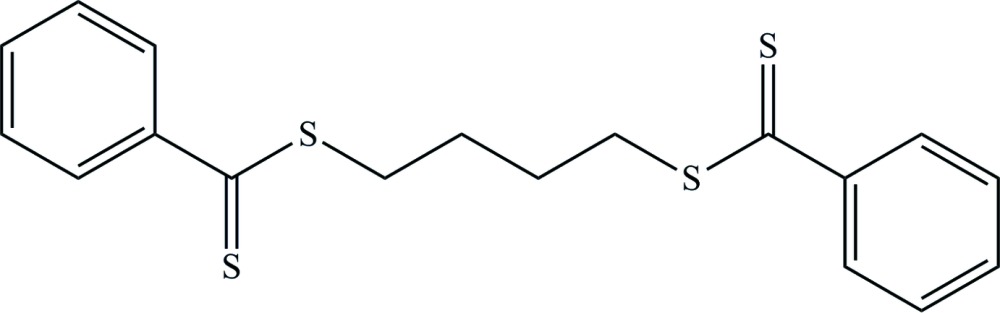


## Experimental

### 

#### Crystal data

C_18_H_18_S_4_
*M* *_r_* = 362.56
Monoclinic, 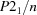

*a* = 11.0205 (6) Å
*b* = 7.2535 (5) Å
*c* = 11.3090 (7) Å
β = 110.805 (2)°
*V* = 845.06 (9) Å^3^
*Z* = 2
Cu *K*α radiation
μ = 5.09 mm^−1^
*T* = 173 K
0.40 × 0.20 × 0.01 mm

#### Data collection

Bruker APEXII CCD area-detector diffractometer
Absorption correction: multi-scan (*SADABS*; Bruker, 2001 ▶) *T*_min_ = 0.235, *T*_max_ = 0.951
4872 measured reflections
1480 independent reflections
1468 reflections with *I* > 2σ(*I*)
*R*_int_ = 0.028

#### Refinement

*R*[*F* ^2^ > 2σ(*F* ^2^)] = 0.037
*wR*(*F* ^2^) = 0.100
*S* = 1.13
1480 reflections
100 parameters
H-atom parameters constrained
Δρ_max_ = 0.35 e Å^−3^
Δρ_min_ = −0.31 e Å^−3^

### 

Data collection: *APEX2* (Bruker, 2007 ▶); cell refinement: *SAINT* (Bruker, 2007 ▶); data reduction: *SAINT*; program(s) used to solve structure: *SHELXS97* (Sheldrick, 2008 ▶); program(s) used to refine structure: *SHELXL97* (Sheldrick, 2008 ▶); molecular graphics: *SHELXTL* (Sheldrick, 2008 ▶) and *Mercury* (Macrae *et al.*, 2006 ▶); software used to prepare material for publication: *SHELXTL*.

## Supplementary Material

Crystal structure: contains datablock(s) I. DOI: 10.1107/S1600536813027529/is5312sup1.cif

Structure factors: contains datablock(s) I. DOI: 10.1107/S1600536813027529/is5312Isup2.hkl

Supplementary material file. DOI: 10.1107/S1600536813027529/is5312Isup3.cml

Additional supplementary materials:  crystallographic information; 3D view; checkCIF report

## Figures and Tables

**Fig. 1.**
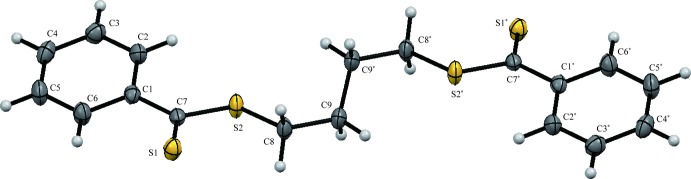
Molecular structure of S,S'-butane-1,4-diyl bis(benzenecarbodithioate) (4DBS4). Displacement ellipsoids are drawn at the 50% probability level.

**Fig. 2.**
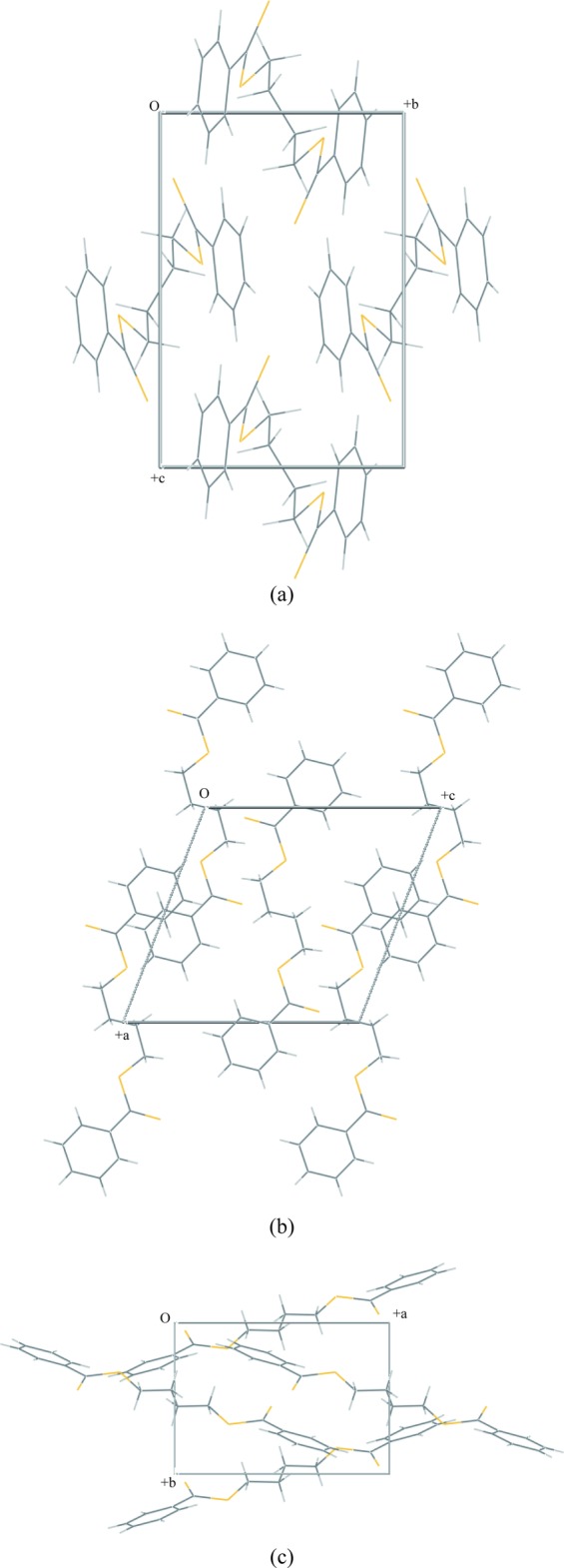
Packing diagrams of 4DBS4, viewed down the a (a), b (b), and c (c) axes.

## supplementary crystallographic information

### 1. Comment

The aromatic polyesters, [–O(CH_2_)*_n_*O(C=O)C_6_H_4_(C=O)-]*_x_* (*n* = 2– 4), have been mass-produced and used as fibers, films, bottles, and engineering plastics. In a series of our studies, we have investigated conformational characteristics and configurational properties of their analogs, [–X(CH_2_)*_n_*X(C=Y)C_6_H_4_(C=Y)-]*_x_*, that is, polythioesters (X = S, Y = O, abbreviated herein as P*n*TS_2_) and polydithioesters (X = Y = S, P*n*TS_4_). As model compounds of P*n*TS_2_ and P*n*TS_4_, we have adopted oligomethylenedithiobenzoate (*n*DBS_2_) and oligomethylenetetrathiobenzoate (*n*DBS_4_), respectively. This paper describes synthesis and X-ray diffraction analysis of one of them, 4DBS_4_.

Figure 1 shows the molecular structure of 4DBS_4_. The S—CH_2_—CH_2_—CH_2_—CH_2_—S bonds lie in the *trans*– *gauche*^+^–*trans*–*gauche*^-^–*trans* (*tg*^+^*tg*^-^*t*) conformation. On the other hand, 4DBS_2_, a model of P4TS_2_, crystallizes to form the *g*^+^*tttg*^-^ conformation (Abe & Sasanuma, 2013). In general, the S—CH_2_ single bond prefers the *gauche* state (Sawanobori *et al.*, 2001; Sasanuma *et al.*, 2002). For instance, the crystalline 2DBS_4_ molecule adopts the *g*^+^*tg*^-^ conformation in the S—CH_2_–CH_2_—S linkage (Abe *et al.*, 2011). By contraries, the two S—CH_2_ bonds of 4DBS_4_ were found here to be in the *trans* conformation. Our molecular orbital calculations at the MP2/6–311+G(2 d,p)//B3LYP/6–311+G(2 d,p) level for gaseous 4DBS_4_ yielded free energies (relative to the all-*trans* state) of the two conformers: 0.49 kcal mol^-1^ (*tg*^+^*tg*^-^*t*) and -0.86 kcal mol^-1^ (*g*^+^*tttg*^-^). Therefore, 4DBS_4_ is not allowed to crystallize in the most stable conformation.

In differential scanning calorimetric measurements, a 4DBS_4_ sample, which was recrystallized from methanol, exhibited only one endothermic peak at 68 °C on heating, whereas its melt-crystallized sample showed two endothermic peaks at 48 and 68 °C. The former and latter samples yielded powder X-ray diffraction patterns different from each other.

Interestingly, *n*DBS_4_'s (*n* = 2, 3, 4, and 5) show odd-even effects in melting; 2DBS_4_ and 4DBS_4_, respectively, melt at 109 and 68 °C, whereas 3DBS_4_ and 5DBS_4_ are liquid at room temperature but exhibit grass transitions at -51 °C (*n* = 3) and -54 °C (*n* = 5).

### 2. Experimental

Piperidinium dithiobenzoate (1.26 g, 5.3 mmol) was prepared according to the literature (Kato *et al.*, 1973). Dibromobutane (0.54 g, 2.5 mmol) was added dropwise into piperidinium dithiobenzoate dissolved in dimethylformamide (DMF, 15 ml) and then stirred for 8 h under nitrogen atmosphere. The reaction mixture was diluted with a mixture of ethyl acetate and n-hexane (1:4 in volume) and washed thrice with water, and the organic layer was dried overnight over anhydrous magnesium sulfate. The solution was condensed, dissolved in a toluene/n-hexane mixture (1:2 in volume), and fractionated by silica-gel chromatograph (*R*_f_ = 0.3–0.5). The collected fractions were condensed and recrystallized from a methanol/n-hexane mixture (1:1 in volume) to yield 4DBS_4_ (0.37 g, 41%).

The product was dissolved in chloroform in an open vessel. The vessel was placed in a larger one containing n-hexane, a poor solvent for 4DBS_4_, to facilitate precipitation of crystals by vapor diffusion of n-hexane into the chloroform solution.

### 3. Refinement

All C—H hydrogen atoms were geometrically positioned with C—H = 0.95 and 0.99 Å for the aromatic and methylene groups, respectively, and refined as riding by *U*_iso_(H) = 1.2 *U*_eq_(C).

### Crystal data

**Table:**

C_18_H_18_S_4_	*F*(000) = 380
*M**_r_* = 362.56	*D*_x_ = 1.425 Mg m^−^^3^
Monoclinic, *P*2_1_/*n*	Melting point: 341 K
Hall symbol: -P 2yn	Cu *K*α radiation, λ = 1.54178 Å
*a* = 11.0205 (6) Å	Cell parameters from 5003 reflections
*b* = 7.2535 (5) Å	θ = 4.8–67.8°
*c* = 11.3090 (7) Å	µ = 5.09 mm^−^^1^
β = 110.805 (2)°	*T* = 173 K
*V* = 845.06 (9) Å^3^	Plate, pink
*Z* = 2	0.40 × 0.20 × 0.01 mm

### Data collection

**Table:**

Bruker APEXII CCD area-detector diffractometer	1480 independent reflections
Radiation source: Bruker TXS fine-focus rotating anode	1468 reflections with *I* > 2σ(*I*)
Bruker Helios multilayer confocal mirror monochromator	*R*_int_ = 0.028
Detector resolution: 8.333 pixels mm^-1^	θ_max_ = 68.1°, θ_min_ = 4.8°
φ and ω scans	*h* = −13→13
Absorption correction: multi-scan (*SADABS*; Bruker, 2001)	*k* = −8→7
*T*_min_ = 0.235, *T*_max_ = 0.951	*l* = −13→13
4872 measured reflections	

### Refinement

**Table:**

Refinement on *F*^2^	Primary atom site location: structure-invariant direct methods
Least-squares matrix: full	Secondary atom site location: difference Fourier map
*R*[*F*^2^ > 2σ(*F*^2^)] = 0.037	Hydrogen site location: inferred from neighbouring sites
*wR*(*F*^2^) = 0.100	H-atom parameters constrained
*S* = 1.13	*w* = 1/[σ^2^(*F*_o_^2^) + (0.0393*P*)^2^ + 1.0478*P*] where *P* = (*F*_o_^2^ + 2*F*_c_^2^)/3
1480 reflections	(Δ/σ)_max_ < 0.001
100 parameters	Δρ_max_ = 0.35 e Å^−^^3^
0 restraints	Δρ_min_ = −0.31 e Å^−^^3^

### Special details

**Table:**

Geometry. All e.s.d.'s (except the e.s.d. in the dihedral angle between two l.s. planes) are estimated using the full covariance matrix. The cell e.s.d.'s are taken into account individually in the estimation of e.s.d.'s in distances, angles and torsion angles; correlations between e.s.d.'s in cell parameters are only used when they are defined by crystal symmetry. An approximate (isotropic) treatment of cell e.s.d.'s is used for estimating e.s.d.'s involving l.s. planes.
Refinement. Refinement of *F*^2^ against ALL reflections. The weighted *R*-factor *wR* and goodness of fit *S* are based on *F*^2^, conventional *R*-factors *R* are based on *F*, with *F* set to zero for negative *F*^2^. The threshold expression of *F*^2^ > σ(*F*^2^) is used only for calculating *R*-factors(gt) *etc*. and is not relevant to the choice of reflections for refinement. *R*-factors based on *F*^2^ are statistically about twice as large as those based on *F*, and *R*-factors based on ALL data will be even larger.

### Fractional atomic coordinates and isotropic or equivalent isotropic displacement parameters (Å^2^)

**Table:**

	*x*	*y*	*z*	*U*_iso_*/*U*_eq_	
C1	−0.0082 (2)	0.2157 (3)	0.3798 (2)	0.0226 (5)	
C2	0.0225 (2)	0.2350 (4)	0.5097 (2)	0.0271 (5)	
H2	0.1068	0.2029	0.5658	0.033*	
C3	−0.0686 (2)	0.3004 (4)	0.5579 (2)	0.0309 (5)	
H3	−0.0463	0.3138	0.6467	0.037*	
C4	−0.1921 (2)	0.3461 (4)	0.4770 (3)	0.0314 (6)	
H4	−0.2547	0.3904	0.5100	0.038*	
C5	−0.2237 (2)	0.3270 (4)	0.3480 (3)	0.0347 (6)	
H5	−0.3085	0.3581	0.2923	0.042*	
C6	−0.1329 (2)	0.2628 (4)	0.2992 (2)	0.0300 (5)	
H6	−0.1556	0.2508	0.2103	0.036*	
C7	0.0885 (2)	0.1450 (3)	0.3270 (2)	0.0222 (5)	
C8	0.3424 (2)	0.0498 (4)	0.3534 (2)	0.0288 (5)	
H8A	0.3123	−0.0796	0.3375	0.035*	
H8B	0.3313	0.1080	0.2710	0.035*	
C9	0.4854 (2)	0.0543 (4)	0.4385 (2)	0.0270 (5)	
H9A	0.5121	0.1841	0.4594	0.032*	
H9B	0.5381	0.0031	0.3913	0.032*	
S1	0.04887 (5)	0.05242 (9)	0.18505 (5)	0.0286 (2)	
S2	0.24732 (5)	0.17228 (9)	0.42927 (5)	0.0278 (2)	

### Atomic displacement parameters (Å^2^)

**Table:**

	*U*^11^	*U*^22^	*U*^33^	*U*^12^	*U*^13^	*U*^23^
C1	0.0177 (10)	0.0217 (12)	0.0276 (11)	−0.0011 (9)	0.0072 (9)	0.0005 (9)
C2	0.0197 (11)	0.0330 (13)	0.0271 (11)	0.0003 (10)	0.0064 (9)	0.0023 (10)
C3	0.0304 (12)	0.0364 (14)	0.0286 (12)	−0.0004 (11)	0.0136 (10)	−0.0013 (11)
C4	0.0255 (12)	0.0310 (14)	0.0429 (14)	0.0014 (10)	0.0186 (11)	−0.0034 (11)
C5	0.0189 (11)	0.0430 (16)	0.0389 (14)	0.0067 (11)	0.0063 (10)	−0.0015 (12)
C6	0.0210 (11)	0.0391 (15)	0.0269 (11)	0.0026 (10)	0.0047 (9)	−0.0026 (10)
C7	0.0169 (10)	0.0228 (12)	0.0255 (11)	0.0010 (8)	0.0058 (8)	0.0044 (9)
C8	0.0178 (11)	0.0406 (15)	0.0283 (11)	0.0071 (10)	0.0087 (9)	−0.0003 (10)
C9	0.0172 (11)	0.0335 (14)	0.0312 (12)	0.0040 (9)	0.0097 (9)	0.0022 (10)
S1	0.0211 (3)	0.0387 (4)	0.0246 (3)	−0.0006 (2)	0.0062 (2)	−0.0052 (2)
S2	0.0143 (3)	0.0394 (4)	0.0271 (3)	0.0035 (2)	0.0040 (2)	−0.0065 (2)

### Geometric parameters (Å, º)

**Table:**

C1—C2	1.392 (3)	C6—H6	0.9500
C1—C6	1.395 (3)	C7—S1	1.649 (2)
C1—C7	1.487 (3)	C7—S2	1.732 (2)
C2—C3	1.386 (4)	C8—C9	1.527 (3)
C2—H2	0.9500	C8—S2	1.807 (2)
C3—C4	1.383 (4)	C8—H8A	0.9900
C3—H3	0.9500	C8—H8B	0.9900
C4—C5	1.381 (4)	C9—C9^i^	1.530 (5)
C4—H4	0.9500	C9—H9A	0.9900
C5—C6	1.384 (3)	C9—H9B	0.9900
C5—H5	0.9500		
			
C2—C1—C6	118.6 (2)	C1—C6—H6	119.8
C2—C1—C7	121.2 (2)	C1—C7—S1	123.48 (16)
C6—C1—C7	120.2 (2)	C1—C7—S2	113.01 (16)
C3—C2—C1	120.7 (2)	S1—C7—S2	123.51 (13)
C3—C2—H2	119.6	C9—C8—S2	109.43 (16)
C1—C2—H2	119.6	C9—C8—H8A	109.8
C4—C3—C2	120.1 (2)	S2—C8—H8A	109.8
C4—C3—H3	120.0	C9—C8—H8B	109.8
C2—C3—H3	120.0	S2—C8—H8B	109.8
C5—C4—C3	119.6 (2)	H8A—C8—H8B	108.2
C5—C4—H4	120.2	C8—C9—C9^i^	113.5 (2)
C3—C4—H4	120.2	C8—C9—H9A	108.9
C4—C5—C6	120.5 (2)	C9^i^—C9—H9A	108.9
C4—C5—H5	119.7	C8—C9—H9B	108.9
C6—C5—H5	119.7	C9^i^—C9—H9B	108.9
C5—C6—C1	120.4 (2)	H9A—C9—H9B	107.7
C5—C6—H6	119.8	C7—S2—C8	104.20 (11)
			
C6—C1—C2—C3	−0.3 (4)	C2—C1—C7—S1	158.30 (19)
C7—C1—C2—C3	−179.8 (2)	C6—C1—C7—S1	−21.2 (3)
C1—C2—C3—C4	0.5 (4)	C2—C1—C7—S2	−22.3 (3)
C2—C3—C4—C5	−0.3 (4)	C6—C1—C7—S2	158.2 (2)
C3—C4—C5—C6	−0.1 (4)	S2—C8—C9—C9^i^	66.7 (3)
C4—C5—C6—C1	0.3 (4)	C1—C7—S2—C8	172.54 (17)
C2—C1—C6—C5	−0.1 (4)	S1—C7—S2—C8	−8.11 (19)
C7—C1—C6—C5	179.4 (2)	C9—C8—S2—C7	−176.88 (17)

Symmetry code: (i) −*x*+1, −*y*, −*z*+1.
